# Undercounting diagnoses in Australian general practice: a data quality study with implications for population health reporting

**DOI:** 10.1186/s12911-024-02560-w

**Published:** 2024-06-05

**Authors:** Rachel Canaway, Christine Chidgey, Christine Mary Hallinan, Daniel Capurro, Douglas IR Boyle

**Affiliations:** 1 Department of General Practice & Primary Care, Faculty of Medicine, Dentistry & Health Sciences, Health & Biomedical Research Information Technology Unit (HaBIC R2), The University of Melbourne, Level 4, Medical Building (BN181), Grattan Street, Melbourne, VIC 3010 Australia; 2 Centre for the Digital Transformation of Health, Faculty of Medicine, Dentistry, and Health Sciences, The University of Melbourne, 700 Swanston St, Melbourne, VIC 3010 Australia; 3 Department of General Medicine, The Royal Melbourne Hospital, 300 Grattan St, Melbourne, VIC 3010 Australia

**Keywords:** Australia, Data quality, Data reporting, Data standards, Diagnosis, Primary health care

## Abstract

**Background:**

Diagnosis can often be recorded in electronic medical records (EMRs) as free-text or using a term with a diagnosis code. Researchers, governments, and agencies, including organisations that deliver incentivised primary care quality improvement programs, frequently utilise coded data only and often ignore free-text entries. Diagnosis data are reported for population healthcare planning including resource allocation for patient care. This study sought to determine if diagnosis counts based on coded diagnosis data only, led to under-reporting of disease prevalence and if so, to what extent for six common or important chronic diseases.

**Methods:**

This cross-sectional data quality study used de-identified EMR data from 84 general practices in Victoria, Australia. Data represented 456,125 patients who attended one of the general practices three or more times in two years between January 2021 and December 2022. We reviewed the percentage and proportional difference between patient counts of coded diagnosis entries alone and patient counts of clinically validated free-text entries for asthma, chronic kidney disease, chronic obstructive pulmonary disease, dementia, type 1 diabetes and type 2 diabetes.

**Results:**

Undercounts were evident in all six diagnoses when using coded diagnoses alone (2.57–36.72% undercount), of these, five were statistically significant. Overall, 26.4% of all patient diagnoses had not been coded. There was high variation between practices in recording of coded diagnoses, but coding for type 2 diabetes was well captured by most practices.

**Conclusion:**

In Australia clinical decision support and the reporting of aggregated patient diagnosis data to government that relies on coded diagnoses can lead to significant underreporting of diagnoses compared to counts that also incorporate clinically validated free-text diagnoses. Diagnosis underreporting can impact on population health, healthcare planning, resource allocation, and patient care. We propose the use of phenotypes derived from clinically validated text entries to enhance the accuracy of diagnosis and disease reporting. There are existing technologies and collaborations from which to build trusted mechanisms to provide greater reliability of general practice EMR data used for secondary purposes.

**Supplementary Information:**

The online version contains supplementary material available at 10.1186/s12911-024-02560-w.

## Background

The secondary use of clinical and administrative data recorded in electronic medical records (EMRs), by healthcare providers, governments, agencies and researchers, is common in Australia and elsewhere [1]. Secondary use of clinical data refers to use of these data for purposes other than providing direct patient care, such as for audit and monitoring, safety, pay-for-performance, disease surveillance, research and teaching [2–4]. The quality of clinical data, however, presents on-going health data science challenges, especially when used for secondary purposes [5–9].

In general practice, secondary use of EMR data can be problematic when clinicians record diagnoses and patient histories as free-text rather than using a diagnosis code from a dropdown list [10–12]. Factors that may inhibit the coding of patient diagnoses by clinicians working in Australian general practice include time constraints, lack of sufficient training to correctly code these fields, and insufficient rules or guidelines to direct or enforce coding [11, 13]. Hospitals employ clinical coders to correctly code patient records for funding purposes, but this is not the case for general practitioners in primary care. Even in settings where professional coders are employed to code patient records there are still many barriers that limit the capture of quality clinical and administrative health data [8, 14, 15].

Barriers to high quality data include incomplete records, lack of standardisation in data capture systems, technological issues and insufficient resources for ongoing training [15]. For example, a United Kingdom study that analysed free and coded text (e.g., history, problem, diagnosis, exam, plan codes) in 65 randomly selected general practice consultations found an average of 6% (range 0–13%) of text was entered as coded data and the remainder as free-text [11]. Furthermore, a study in the United States to validate EMR-derived quality measures found significant undercounting resulting from either incorrectly coded information or information in formats unreadable by automated data methods (i.e., attached letters or reports) [16].

Using data extraction tools to collect and curate clinical data from EMR systems is common [17]. Tools used to calculate quality and performance metrics from community and primary care data EMRs provide a practical approach for assessing and reporting performance and population health related outcomes [2, 18]. Despite progress in the development of natural language processing in medicine over the past 20 years, the current tools tend to rely on coded data only [19].

In the Australian primary care sector 31 Primary Health Networks (PHNs), funded by the Australian Government, coordinate primary health care to improve efficiency, effectiveness and access [20]. The PHNs provide general practices with third party data extraction tools that extract aggregated coded diagnosis data from patient records. PHNs use this for multiple purposes, including planning, reporting quality and performance data to the government, and providing feedback to general practice. PHNs also provide general practices with instructions on how to ‘clean’ free-text entries, by mapping them to coded diagnosis terms that can be included when reporting quality improvement (QI) measures as part of the government Practice Incentive Program (PIP QI) [21, 22].

While testing a new national tool for analysing the quality of Australian health care data repositories, the authors observed the extent of free-text recording in GP EMR systems. The limitations around the use of coded data only for general practice quality and performance metrics have not been thoroughly explored in Australia. This study tested the hypothesis that the use of coded diagnosis data only to generate quality metrics or statistics on the number of patients with specific diagnoses would result in underreporting compared to the use of coded and free-text diagnosis.

## Methods

We analysed de-identified EMR data from The University of Melbourne’s Patron primary care data repository [23, 24] which comprises data extracted using GRHANITE® software installed on computers at general practices that participate in the ‘Data for Decisions’ primary care research network (www.unimelb.edu.au/datafordecisions). The characteristics of the data repository and demographic profile of patients are summarised elsewhere [23].

This study had University of Melbourne Human Research Ethics Committee approval (Ethics ID 1,852,031) and approval from the independent Patron Data Governance Committee to use patient de-identified data from the Patron repository for this research. A University of Melbourne ethics committee approved waiver of patient informed consent is in place as part of the Patron / Data for Decisions program of work (Ethics ID 23,358). All methods were performed in accordance with the relevant guidelines and regulations.

All general practices represented used Best Practice (BP), MedicalDirector (MD) or Zedmed (ZM) clinical software systems. The data analysed was sourced from 84 practices in Victoria, Australia as at 31 December 2022 and was restricted to ‘active patients’ using the Royal Australian College of General Practitioners (RACGP) definition (persons having three or more consultations in the previous two years) [25]. The ‘RACGP active’ designation was used so our output could be comparable to other Australian primary care reporting that frequently align with this definition. The data were housed in a secure, limited access, University of Melbourne secure research environment. Data analyses were carried out using SQL queries using Microsoft SQL Server Management Studio v.18.9.1.

**Fig. 1:**
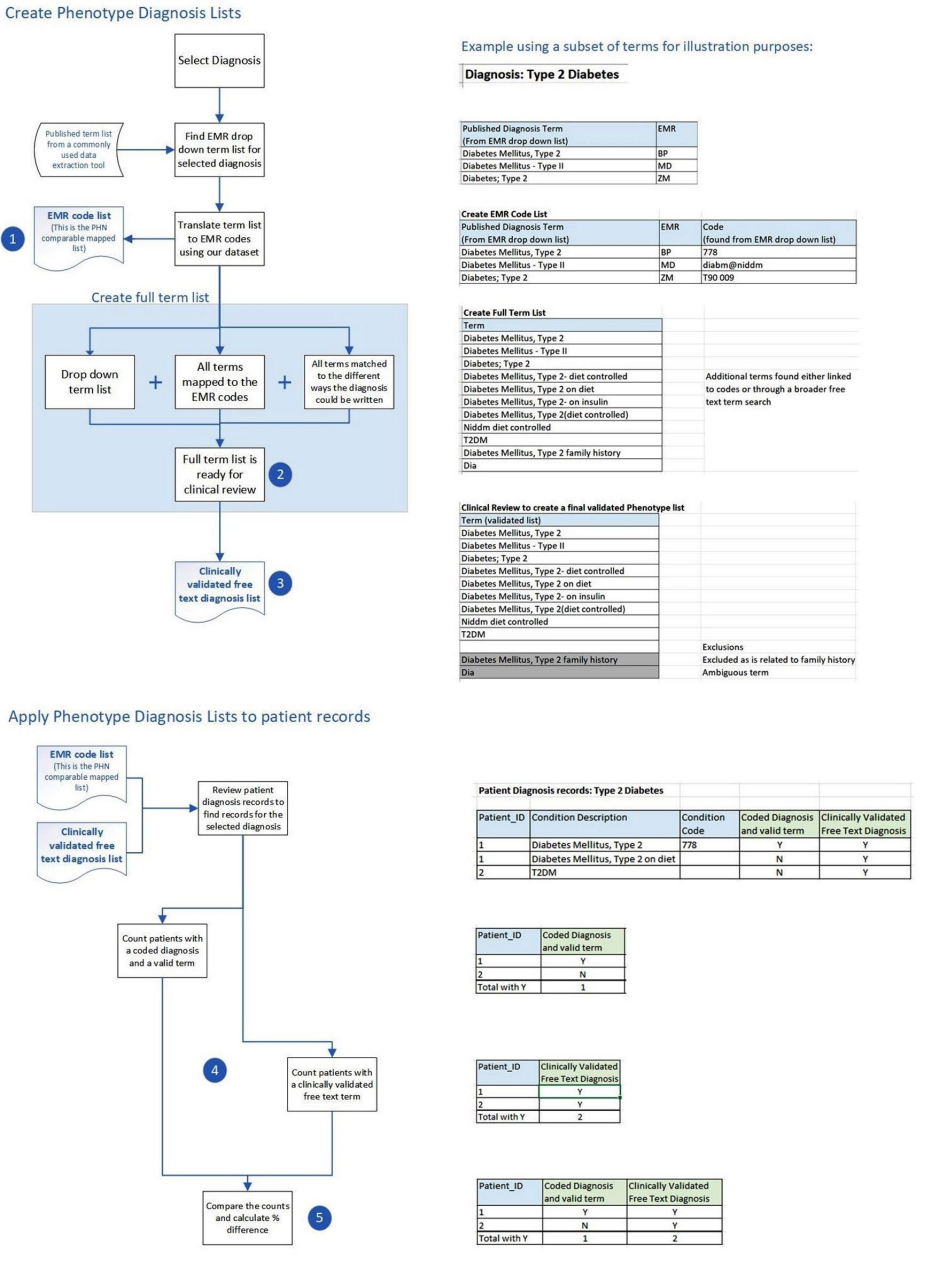
Matching diagnosis codes with validated free-text for count comparison: Best Practice (BP), Medical Director (MD) and Zedmed (ZM) clinical data, using Type 2 diabetes as an example

We analysed six chronic diseases: asthma, chronic kidney disease (CKD), chronic obstructive pulmonary disease (COPD), dementia, type 1 diabetes (T1DM), and type 2 diabetes (T2DM). These are common diseases in general practice and are frequently included in primary care research projects. Since the ‘diagnosis’ field in the included EMR systems can store either coded data or short strings of free text, we created digital phenotypes based on these two data types. We utilised in-house clinical expertise (described at point 3 below) to develop clinically validated diagnosis phenotyping for these diseases specifically to capture the free-text diagnoses. We were then able to compare the counts of coded diagnoses alone with the phenotype clinically validated free-text diagnoses for each disease, as described below and in Fig. 1 (using the example of T2DM):

1. **Create EMR code lists**: We collected a set of EMR dropdown/lookup list diagnosis terms from a commonly used data extraction tool utilised in general practice [26]. With this list of publicly available terms, the corresponding EMR diagnosis codes were translated from our general practice data set. The EMR code list then allowed us to create a mapping comparable to that used by PHNs to generate statistics used for the majority of general practice reporting in Australia.
2. **Create free-text term lists**: Using our general practice data set, we queried patients’ diagnosis histories to create a broad list of possible diagnosis term matches for each disease, including all terms mapped to the diagnosis codes and all terms matched to different ways a diagnosis could be written in free-text (e.g., T2DM or DMT2). Patient diagnosis data tables included past diagnoses recorded prior to the two-year ‘RACGP active’ patient window, as all diagnosis history records were deemed relevant. The historical data period available varied between patients and practices. Only data from the EMR diagnosis field was included in our queries. Data from other fields such as reason for visit and reason for prescription was not included. Free-text diagnoses in clinical notes are not included in the Patron repository and so could not be used to create the free term list.
3. **Clinical review of free-text term lists**: The free-text list was subject to expert clinical review, by academic medical doctors at the University of Melbourne’s Department of General Practice and Primary Care, to validate free-text terms that would mean a diagnosis had been made and to remove free-text terms that were ambiguous or not related to the diagnosis. Since the strings of text stored in this field are short, without complex linguistic structure or negations, we reviewed them manually and did not use any NLP methods. The resulting list of validated diagnosis terms was then used to find the counts. This clinical review additionally found that a small number of terms were mapped incorrectly to diagnosis codes, which would result in false positive diagnosis counts (incorrect mapping can occur when practices use inbuilt EMR tools to do their own mapping of free-text i.e., the code is wrong, not the free-text). All incorrectly mapped terms were excluded to avoid counting false positive diagnoses in our comparison; the number excluded are reported in the results below.
4. **Compare counts**: The counts across the six diagnoses were calculated, first for patients with a coded diagnosis (which by default have a corresponding clinically validated term that is displayed in the EMR lookup list i.e., it is not possible to have a code only), and second for patients with a clinically validated free-text diagnosis term (with or without the presence of a corresponding diagnosis code). We ensured that patients were only counted once in each calculation. For example, if a patient had multiple term/code diagnosis matches they would only be counted once, and if a patient had a coded diagnosis they would be included in that calculation even if they had additional free-text matches.
5. **Calculate the percentage difference**: The percentage difference between the counts generated at step 4 were calculated (coded diagnosis versus clinically validated free-text diagnosis with or without a coded diagnosis). We used the z-test of difference between two proportions to test for significance.

## Results

The data from 84 general practices contained 456,125 patients who met the RACGP’s definition of ‘active’ patient [25]. Table 1 illustrates the variance among those ‘RACGP active’ patients across the six studied chronic diseases when counted via coded diagnoses alone (as per reporting to government) versus clinically validated free-text AND coded diagnoses. For all diseases, aggregated patient counts based on coded diagnoses only (column A) were fewer than counts that included clinically validated free-text diagnoses (column B). Of all diagnoses across the six diseases, 26.4% were uncoded. The statistically significant undercount differences ranged from 2.57% for Type 2 diabetes to 36.72% for asthma. The difference between count methods for Type 1 diabetes was not statistically significant. The difference between two proportions (z-test), i.e., between coded diagnoses only versus clinically validated free-text diagnoses populations across the six indicators, was significant (*p*-value < 0.001 for population *N* = 456,125).

Excluded from the data shown in Table 1 column A, due to terms found to be incorrectly mapped to codes (Methods Step 3) were: Asthma *n* = 46 (0.10%), CKD *n* = 1 (0.01%), COPD *n* = 9 (0.12%), Dementia *n* = 57 (2.58%), type 1 diabetes *n* = 1 (0.05%) and type 2 diabetes *n* = 3 (0.01%).

**Table 1:** Variance in counts between coded only and free-text plus coded diagnoses in general practice medical records, *N* = 456,125 ‘active’ patients*

	A	B	C	D	E	F
Disease	Patients with a coded diagnosis	Patients with a clinically validated free-text diagnosis (with or without a code)	Patients with a clinically validated free-text diagnosis but no coded diagnosis. (B-A = undercount)	% Undercount	Z value	*P* value
Asthma	46,853	74,038	27,185	36.72	156.18	< 0.001
Chronic kidney disease	8303	10,721	2418	22.55	17.72	< 0.001
Chronic obstructive pulmonary disease	7774	9573	1799	18.79	13.79	< 0.001
Dementia	2153	2525	372	14.73	5.45	< 0.001
Type 1 diabetes	2033	2112	79	3.74	1.23	0.219
Type 2 diabetes	23,264	23,877	613	2.57	2.90	0.004
All diagnosis types **	90,380	122,846	32,466	26.4	80.32	< 0.001

* Active as per the RACGP definition, i.e. a patient who has had a general practice consultation three or more times in the past two years [25], which in this instance was between 1 January 2021 and 31 December 2022

** Patients are counted only once per disease, but the total for ‘All diagnosis types’ will include patients multiple times if they have more than one disease

**Fig. 2:**
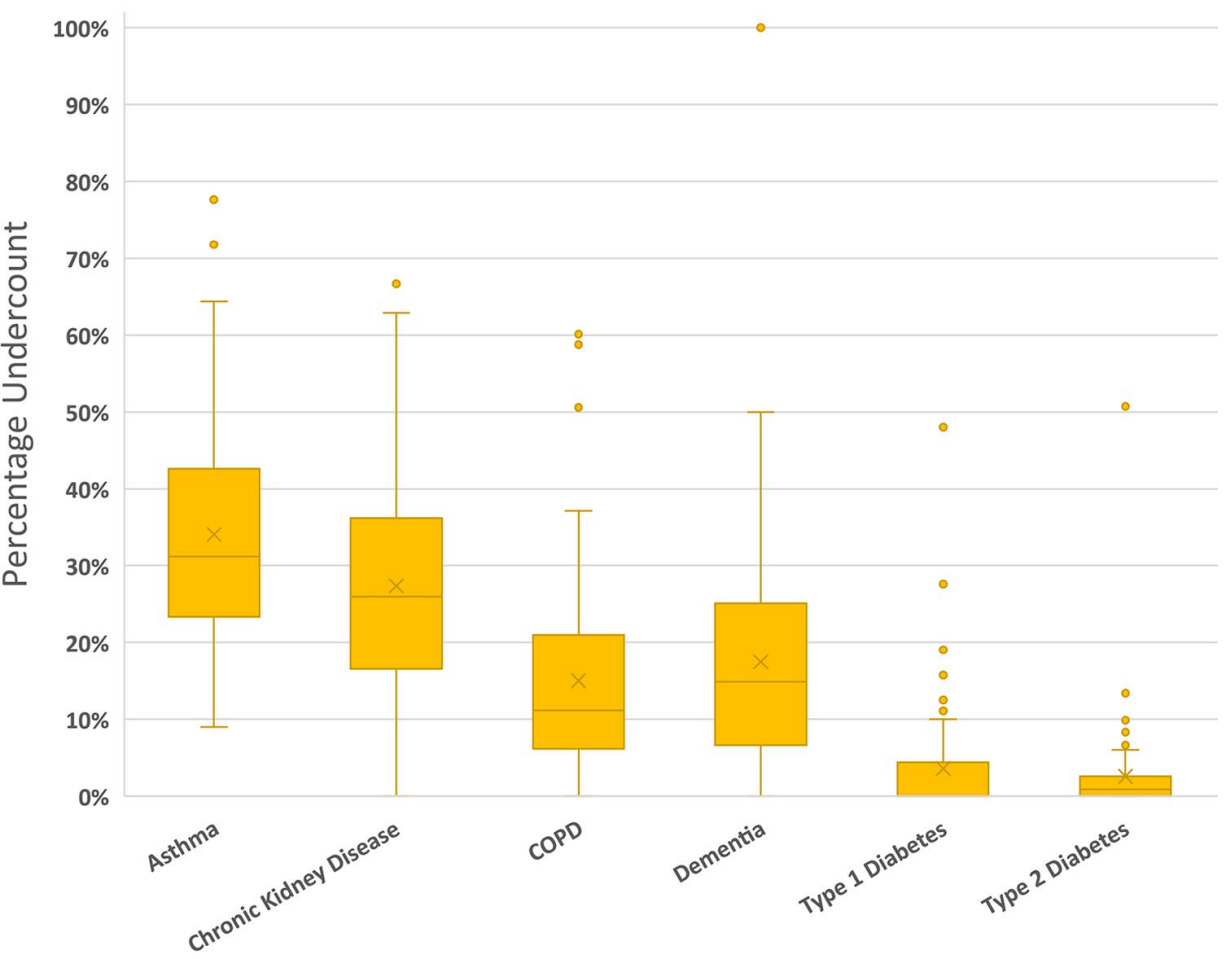
The percentage difference in undercounting for each diagnosis across the 84 general practices. COPD = Chronic Obstructive Pulmonary Disease

We also reviewed the variation of undercount across practices. Figure 2 and the supplementary Table 1 highlight the wide variation between practices in capture of coded diagnoses, from zero undercount (for all conditions excepting asthma) to very high levels of undercount. The table quantifies the relatively small numbers of patients diagnosed with dementia and/or T1DM per practice (median *n* = 20 max 115 and median *n* = 24 max *n* = 81 respectively, coded and free-text). For practices with very small numbers of patients with a particular diagnosis, a missed coded diagnosis could result in a very high undercount; for example, the practice with 100% undercount of dementia had one patient with dementia recorded as free-text and none coded (i.e., resulting in 100% undercount). The T1DM outlier practice (48.0% variance) had 13 coded out of 25 free-text and coded diagnoses.

## Discussion

Our analysis of de-identified data from Australian general practice patient records highlights that relying solely on coded diagnoses, as commonly done in PHN and national healthcare statistics, is likely to result in statistically significant undercounting of certain diseases. Among our sample of 456,125 ‘RACGP active’ general practice patients, when using clinically validated free-text diagnoses, we found 122,846 counts of the six diseases compared with 90,380 counts when using coded diagnoses only (where one person is counted once per disease, but one person will be included multiple times if they have more than one of the diseases of interest). Therefore, 26.4% (*n* = 32,466) of counts that did not contain a coded diagnosis would not be included in routine reporting such as PIP QI which specifies use of ‘RACGP active’ patient counts [22]. Further, we found (and excluded) instances where terms were incorrectly mapped to codes leading to slightly raised diagnoses counts. These errors could have resulted from human error when manually mapping free-text terms to codes using tools built into the EMR system (i.e., CHD to CKD or vice versa).

There is a high level of variation in percentage of undercount between practices (depicted by the boxplots at Fig. 2) which indicates that some practices are more consistently inputting coded diagnoses. Decerning the reasons for these differences between practices is beyond the scope of this data review. However, the undercounts for type 1 diabetes and type 2 diabetes were small, indicating that coding for these diseases in general practice tends to be present. This may reflect success of PIP QI that, for several years, has included focus on accurate coding for diabetes [22] The same consistency is not present for dementia, CKD, COPD, and asthma which have not had the same level of incentive for practices to spend time accurately coding.

In Australia there are no ‘standard’ digital phenotypes for medical diagnoses. A potential problem arises with Australia’s PHN-managed general practice PIP QI program which is designed to incentivise improvements in general practice quality and performance [22]. There are two main organisations/systems providing PHNs with data about the indicators used for PIP QI, and the program also allows practices to submit their data to PHN’s using other third-party tools. Without standard phenotype definitions, statistics generated from the different systems may not be comparable, and as we have shown, the exclusion of clinically validated free-text diagnoses can lead to significant undercounting.

Despite efficient look-up mechanisms and initiatives that encourage GPs to clinically code diagnoses and systematically clean practice data, the level of free-text recording we found demonstrates GPs continue to use free-text and are likely to continue to record in this way [13]. International evidence indicates doctors need to record free-text to accurately capture the context of their observations [6]. Alternatives to free-text documentation of discrete concepts—such as diagnosis and reason for visit—include the adoption of interface terminologies and terminology servers. With interface terminologies, clinicians can select concepts meaningful to their daily practice which have, in turn, been mapped to standard terminologies like SNOMED-CT [27]. These interface terminologies have shown to improve clinical documentation and data quality [28]. Terminology servers allow clinicians to input information using free-text, and in real time, map the terms to standard terminologies, to improve data quality [29]. Both approaches could be considered for general practice EMRs to improve capture of coded fields (such as diagnoses) without disrupting clinical workflow. A third approach could include the use of NLP [30] or, most recently, large language models for coding clinical episodes [31] but as most multi-class classifiers, their overall accuracy remains lower than required for widespread adoption, though the landscape is quickly changing.

Reliance on coded data practices to create practice-level estimates of counts is more adequate for some diagnoses (e.g., diabetes) than others (e.g. asthma), but the findings also demonstrated that using EMR data from GP practices to create population-level estimates requires data-linkage of patient records across time to create more accurate population morbidity profiles. Nonetheless, accurate data are essential to clinical care, population health planning and research; the statistics that underpin such activities must be trusted. The undercounting of CKD, for example: 22.6% of patients with a clinically validated free-text diagnosis excluded from reporting, may contribute to the 17% of Australian patients with CKD being referred late which is associated with poorer health outcomes [32].

If governments mandated clinical phenotype definitions for general practice reporting, this would increase data accuracy to better reflect actual population incidence. This is especially important for where outcome-based target and performance payment systems are in place. As a part of the solution for Australia, we propose a national community of practice charged with representing the interests of all stakeholders to work in a national, collaborative manner to establish standard phenotype definitions that incorporate clinically validated free-text and curated mappings to standard terms.

A technical solution to the curation of standardised phenotype definitions is in development by the Australian Research Data Commons as an open-access, Australian tool able to host terminologies/definitions on behalf of communities of practice. This development is in conjunction with The University of Melbourne and the Commonwealth Scientific and Industrial Research Organisation (CSIRO) and is being developed under the auspices of the Australian Health Research Alliance’s (AHRA) national systems level initiative–the Transformational Data Collaboration (AHRA TDC) [33]. This technical solution utilises existing national infrastructure (Ontoserver, a terminology server [34]) being developed by the Australian Digital Health Agency and CSIRO.

## Limitations

We are likely to have missed free-text terms used by general practitioners, for example, we would not have captured all mistaken spelling of the diagnoses studied. Also, by including only RACGP defined ‘active’ patients, persons who visit a GP fewer than three times in two years were excluded. Given the chronic nature of the diseases reviewed it is likely that many persons would fall into the ‘RACGP active’ patient classification; however, those who visit less frequently, or ‘shop’ between more than one GP practice for their care, are likely to have been excluded. As previously noted, we did not analyse free-text within the EMR found in letters, reports, and clinical notes, thus limiting the ability to calculate a sensitivity and specificity of the digital phenotype used. Due to the reasons above, our figures of undercount are likely conservative.

Our study did not examine the impact of local nor national quality improvement and incentive programs on practice level capture of coded fields. A longitudinal study with findings temporally matched to the implementation of data QI programs could look for correlation between such programs and changes in rates of capture of coded data. A new study using multilevel analysis could further explore reasons for continued use of free-text instead of coded diagnosis. Such a study ought to examine general practitioner’s data capture preferences, the clinical terminology system implemented by the underlying EMR system (noting that there are multiple systems in use with some more popular than others), whether the practice has changed EMR system (previously coded data may migrate as text only), how actively practices participate in data QI activities, practice size and patient demographics.

## Conclusion

We have shown that in Australia, the use of general practice coded diagnoses for reporting, to the exclusion of clinically validated free-text diagnoses, can lead to a statistically significant degree of diagnosis undercounting (e.g., 14.7% undercounting of dementia, 18.8% COPD, 22.6% CKD, 36.7% asthma). Failure to account for free text diagnostic data entry impacts the ability to initiate computerised risk assessment and patient recall, impacts the ability of a practice to manage their at-risk populations and leads to the underestimation of significant conditions such as those described above. This significantly impacts population health planning and policy setting. These shortcomings can result in delayed patient treatment and the associated costs to the health system of more advanced disease states. While enhanced coding is always advantageous, textual data entry is a part of the real world. We propose that the validity of using phenotypes derived from clinically validated free-text data entries should be further examined on a national level to make disease reporting more accurate. A national community of practice can guide the building of an open, national capability to reach consensus on phenotype definitions. Existing technologies and collaborations can be utilised to provide greater reliability of general practice EMR data for secondary healthcare purposes.

### Electronic supplementary material

Below is the link to the electronic supplementary material.

Supplementary Material 1

## Data Availability

The general practice electronic medical record dataset supporting the conclusions of this article is from the Patron primary care data repository https://melbourne.figshare.com/articles/dataset/PATRON_Primary_Care_Research_Data_Repository/7653485. The dataset is not exportable outside of the secure Patron data enclave. The datasets generated and analysed during the current study are not publicly available because although de-identified, Patron data are considered sensitive, and so are accessible only within the secure research environment with Patron Data Governance Committee and Ethics Committee approvals.

## Abbreviations

AHRA TDC: Australian Health Research Alliance – Transformational Data Collaboration
BP: Best Practice
CKD: Chronic Kidney Disease
COPD: Chronic Obstructive Pulmonary Disease
EMR: Electronic Medical Record
GP: General Practitioner
MD: MedicalDirector
QI: Quality Improvement
PHN: Primary Health Network
PIP-QI: Practice Incentive Program – Quality Improvement
RACGP: Royal Australian College of General Practitioners
ZM: Zedmed
