# Supplementary material for: Undercounting diagnoses in Australian general practice: a data quality study with implications for population health reporting
Source: BMC Med Inform Decis Mak. 2024 Jun 5;24:155. doi: 10.1186/s12911-024-02560-w (PMC11151573; doi:10.1186/s12911-024-02560-w)
Supplement: Supplementary file 1 — Supplementary Material 1 [file 12911_2024_2560_MOESM1_ESM.docx]

**Supplementary material**

**Table: Variation in coding across the 84 general practices, N=456,125 ‘active’ patients***

|  | **Asthma** | | | **Chronic Kidney Disease** | | | **Chronic Obstructive Pulmonary Disease** | | | **Dementia** | | | **Type 1 diabetes** | | | **Type 2 diabetes** | | |
| --- | --- | --- | --- | --- | --- | --- | --- | --- | --- | --- | --- | --- | --- | --- | --- | --- | --- | --- |
| **Patient counts  at practice-level** | A  (n) | B  (n) | C  (%) | A  (n) | B  (n) | C  (%) | A  (n) | B  (n) | C  (%) | A  (n) | B  (n) | C  (%) | A  (n) | B  (n) | C  (%) | A  (n) | B  (n) | C  (%) |
| **Minimum** | 25 | 38 | 9.0 | 4 | 9 | 0.0 | 3 | 4 | 0.0 | 0 | 1 | 0.0 | 2 | 2 | 0.0 | 12 | 12 | 0.0 |
| **Maximum** | 2402 | 3589 | 77.6 | 587 | 615 | 66.7 | 531 | 668 | 60.6 | 111 | 115 | 100.0 | 79 | 81 | 48.0 | 1121 | 1184 | 50.7 |
| **Quartile 1** | 311 | 511 | 23.3 | 37 | 55 | 16.6 | 39 | 44 | 6.1 | 8 | 10 | 6.6 | 15 | 15 | 0.0 | 138 | 142 | 0.0 |
| **Median** | 508 | 786 | 31.2 | 87 | 119 | 26.0 | 82 | 99 | 11.2 | 15 | 20 | 14.9 | 23 | 24 | 0.0 | 237 | 247 | 0.9 |
| **Quartile 3** | 735 | 1036 | 42.6 | 141 | 176 | 36.2 | 130 | 157 | 21.0 | 42 | 51 | 25.1 | 31 | 32 | 4.4 | 346 | 365 | 2.6 |
| **Mean** | 571 | 903 | 34.0 | 101 | 131 | 27.4 | 95 | 117 | 15.0 | 26 | 31 | 17.5 | 25 | 26 | 3.6 | 284 | 291 | 2.6 |
| **Total cases across 84 practices**** | 46,853 | 74,038 |  | 8,303 | 10,721 |  | 7,774 | 9,573 |  | 2,153 | 2,525 |  | 2,033 | 2,112 |  | 23,264 | 23,877 |  |

Column A = Patients with a coded diagnosis (practice-level)

Column B = Patients with a clinically validated free-text diagnosis (and/or coded diagnosis, practice-level)

Column C = % undercounts (practice-level % = (patients with a clinically validated free-text diagnosis but no coded diagnosis) / (total patients with clinically validated free-text diagnosis and/or coded diagnosis) x 100). Column C data are used in the Figure 2 Boxplot.

* Active patient as per the RACGP definition, i.e. a patient who has had a general practice consultation three or more times in the past two years, which in this instance was between 1 January 2021 and 31 December 2022 [25]. Note that patients are counted only once per disease but may be included under more than once disease category.

** As per columns A and B in Table 1 of the main manuscript.
